# Strathclyde Minor Groove Binders Bearing Amidine Tail Groups are Effective against Gram-Positive Bacterial Pathogens

**DOI:** 10.1021/acsinfecdis.6c00443

**Published:** 2026-07-14

**Authors:** Charlotte K. Hind, Kaveh Eskandari, Leah M. C. McGee, Rebecca Beveridge, Louise C. Young, Edward Hutchings, Matthew E. Wand, Melanie Clifford, J. Mark Sutton, Fraser J. Scott

**Affiliations:** † 164131 Research and Evaluation, UKHSA Porton Down, Salisbury SP4 0JG, U.K.; ‡ Department of Pure and Applied Chemistry, 3527 University of Strathclyde, Glasgow G1 1XQ, U.K.; § Strathclyde Institute of Pharmacy and Biomedical Sciences, University of Strathclyde, Glasgow G1 1XQ, U.K.; ∥ Institute of Pharmaceutical Science, School of Cancer & Pharmaceutical Science, King’s College London, Franklin-Wilkins Building, 150 Stamford Street, London SE1 9NH, U.K.

**Keywords:** antibacterial, minor groove binder, DNA, Gram-positive

## Abstract

The rise of multidrug-resistant Gram-positive pathogens necessitates new antibacterial agents with mechanisms that are distinct from those of existing therapies. Here, we report the antibacterial activity and mechanistic characterization of two Strathclyde minor groove binders (S-MGBs), S-MGB-234 and S-MGB-235, synthetic DNA binding molecules designed to disrupt essential bacterial processes. These compounds displayed potent in vitro activity against clinically relevant Gram-positive pathogens, including *Staphylococcus aureus* and *Enterococcus*spp. However, potency was less pronounced against *Enterococcus faecalis*. Activity was retained against drug-resistant strains, and reduced susceptibility emerged more slowly during serial passaging than that observed for gentamicin under the conditions tested. Whole-genome sequencing of reduced susceptibility mutants did not identify mutations in canonical DNA targets but instead revealed recurring changes in genes associated with the cell envelope, including norA, fmtA, and cozEb, suggesting that envelope-mediated effects influence compound access rather than direct target modification. Biophysical assays demonstrate strong interactions with AT-rich oligonucleotides and gDNA, consistent with the DNA binding properties previously reported for the S-MGB class. Together, these findings demonstrate that S-MGBs represent a promising class of DNA-targeting antibacterials and provide initial evidence that reduced susceptibility emerges through heterogeneous mechanisms that do not involve obvious modifications of DNA targets.

## Graphical abstract

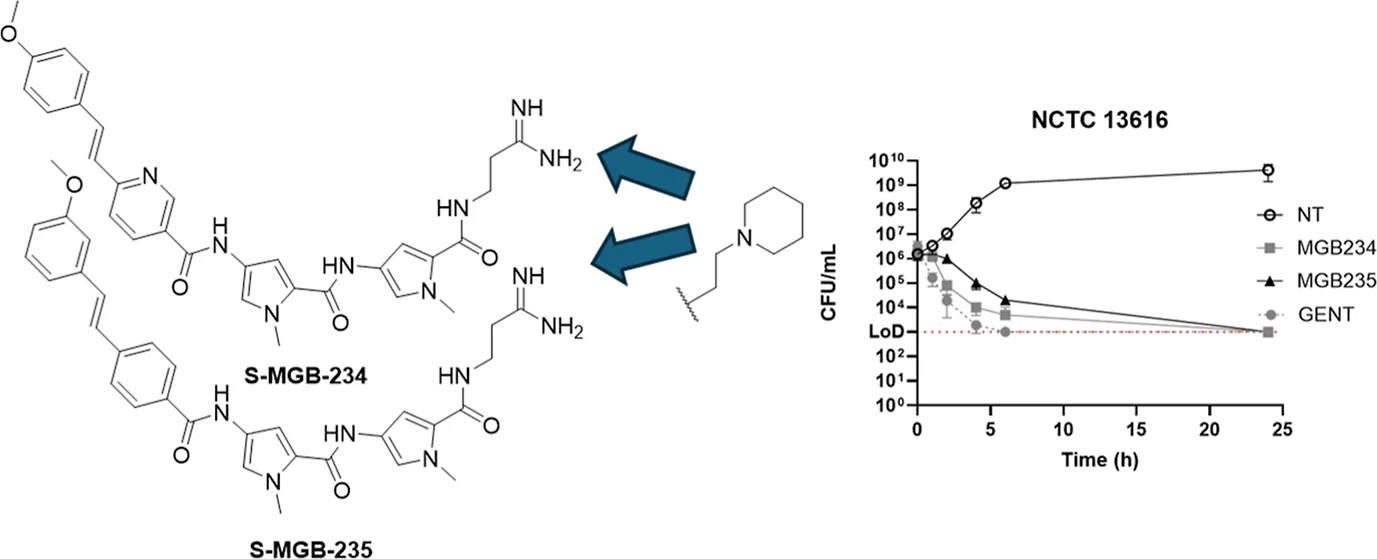

## Background

*Staphylococcus aureus* was the second highest leading cause of the 4.95 million deaths associated with antimicrobial-resistant (AMR) infections in 2019, only outnumbered by *Escherichia coli*.^1^ Of the 1.27 million deaths directly attributable to antimicrobial-resistant infections recorded in 2019, the leading pathogen-drug-resistant combination responsible for these deaths was methicillin-resistant *S. aureus* (MRSA). For both deaths attributable to and deaths associated with AMR, MRSA was the highest increasing cause between 1990 and 2021.^2^ The WHO has categorized MRSA as a “high” priority pathogen in their 2024 Priority Pathogen list for discovery and development of new treatments, based on its levels of multidrug resistance and the resulting global burden in healthcare settings.^3^

Minor groove binders (MGBs) are a class of small molecules that bind to the minor groove of double stranded DNA (dsDNA), causing detrimental effects on a pathogen’s biological processes, such as transcription or action of topoisomerases.^4,5^ Binding to target DNA sequences may cause direct occlusion of a key DNA–protein interaction site, or through indirect topological changes.^6^

There are a variety of structural subclasses of MGB, including diamidines (e.g., diminazene) which have generally been explored scientifically, or used clinically, for antiprotozoal purposes.^7–9^ However, pentamidine, another diamidine, has been investigated in an antibacterial context. Although typically considered as an antifungal or antiprotozoal, it was found to be active in vitro against a number of bacterial pathogens, including *S. aureus*.^10^ More recently, Stokes et al. demonstrated that pentamidine can disrupt the stability of the outer membrane LPS of Gram-negative bacteria allowing for synergistic activity with drugs such as vancomycin, rifampicin, novobiocin, and erythromycin, which are traditionally only effective against Gram-positive pathogens.^11^

The polyamides, exemplified by the natural products distamycin and netropsin, are another structural subclass of the MGB. Strathclyde MGBs (S-MGBs) are a drug discovery platform, developed at the University of Strathclyde, which are derived primarily from the structure of distamycin. Consequently, S-MGBs bind strongly to many AT-rich DNA sequences, like distamycin, embracing a multitargeted anti-infective drug (MTAID) approach.^12^ The MTAID approach is designed to reduce the propensity toward target-based resistance in pathogens. Indeed, this has been demonstrated for the most developed S-MGB, MGB-BP-3, which has successfully completed phase IIa clinical trials for the treatment of CDAD.^13^

Since the discovery and development of MGB-BP-3, the S-MGB class has been shown to have a broad spectrum of activity across different phylogenetic groups of pathogens, namely, bacteria, fungi, viruses, and protozoa.^13–24^ More recently, we have identified that the modification of the tail group of S-MGBs to include an amidine moiety, rather than the morpholine found in MGB-BP-3, can result in significant changes to the biological consequences of these molecules. For example, there is tentative evidence that the amidine moiety results in improved binding to dsDNA while also reducing intracellular accumulation in mammalian cells, the latter of which results in improved selectivity.^25,26^ We have also shown that the most promising S-MGBs to treat animal African trypanosomiasis, caused by the protozoal pathogens *Trypanosoma congolenese* and *T. vivax*, also bear the amidine tail group; here, S-MGB-234 and S-MGB-235 have been shown to be effective in vivo.^15^

Although MGB-BP-3 has progressed well through clinical trials, its target indication is narrow, specifically *Clostrioidies difficile*, due to its optimized physicochemical properties for no systemic absorption following oral administration. Therefore, we have been interested in returning focus to the development of S-MGBs with antibacterial activity that can be used for other infections. Additionally, noting that MGB-BP-3 bears a morpholine tail group, we were specifically interested in understanding the utility of S-MGBs bearing an amidine tail in the antibacterial context, as these are emerging as promising in other anti-infective contexts.

Specifically, in this study, we have investigated the amidine tail group containing S-MGB-234 and S-MGB-235, two compounds that have been shown to be safe and effective in vivo as antiprotozoals.^15^ To better understand the role of the tail group, we have also investigated the morpholine tail group containing analogues of these compounds, S-MGB-2 and S-MGB-1, respectively.

## Results and Discussion

### Amidine Tail Group S-MGBs Retain a Gram-Positive-Selective Antibacterial Profile, with Reduced Cytotoxicity against Mammalian Cells

The activity of S-MGB-1, S-MGB-2, S-MGB-235, and S-MGB-234 was assessed against a panel of ESKAPE pathogens, including the Gram-positives *S. aureus* and *Enterococcus faecalis* and the Gram-negatives *E. coli*, *P. aeruginosa*, *K. pneumoniae*, and *A. baumannii*, by EUCAST methods (Table 1). S-MGB-1 and S-MGB-2 have previously been evaluated for antibacterial activity against *S. aureus* and *E. faecalis*, but the specific strains used were not indicated, and therefore, we included these molecules to allow for a direct comparison.^16^ Cytotoxicity was assessed using a PrestoBlue viability assay against HEK293 cells, first by a restricted concentration screen (Figure S1) and then by full IC_50_ determination for those compounds showing significant reduction in viability in the screen (Table 1).

**Table 1:** MICs against ESKAPE Pathogens and Cytotoxicity against HEK293 Cells

compound	*S*. *Aureus* (ATCC 43300) MIC (μM)	*E*. *faecalis* (ATCC 51299) MIC (μM)	Gram-negative pathogens^a^ MIC (μM)	HEK293 IC_50_ (μM)	selectivity index (HEK293/*S*. *aureus*)	selectivity index (HEK293/*E*. *faecalis*)
1	6.25	6.25	>100	44 ± 2	7	7
235	6.25	12.5	>100	>100	>16	>8
2	1.56	6.25	>100	70 ± 6	45	6
234	6.25	25	>100	>100	>16	>4

a *E. coli* 25922, *K. pneumoniae* 700603, *P. aeruginosa* 27893, and *A. baumannii* 19606.

In line with what is known about the spectrum of antibacterial activity of S-MGBs, all S-MGBs had MICs >100 μM against the Gram-negative pathogens.^27^ MICs against *E. faecalis* were in general slightly higher (≤4-fold) than those against *S. aureus*. There was a small increase in minimum inhibitory concentration (MIC) associated with the modification of the tail group from the morpholine (S-MGB-1, S-MGB-2) to the amidine (S-MGB-235, S-MGB-234), mainly between S-MGB-2 and S-MGB-234 with a 4-fold increase across both Gram-positive organisms. The cytotoxicity is consistently lower for the amidine tail compounds (S-MGB-235, S-MGB-234), which is consistent with data emerging from other S-MGB studies.^15,19,25^ Indeed, for S-MGB-1 and S-MGB-235, the modification to the amidine tail group has resulted in improved selectivity indices. This could be the case for S-MGB-2 and S-MGB-234, but it is masked by the lower limit of the selectivity index being constrained by the upper limit of the HEK293 cytotoxicity assay. In general, we suggest that the modification of the S-MGB tail group to the amidine moiety has improved cytotoxicity while maintaining potency against Gram-positive pathogens.

### S-MGB-234 and S-MGB-235 Are More Potent toward Staphylococci Compared to Enterococci

The preliminary screen against ESKAPE pathogens confirmed potency against Gram-positive organisms only; therefore, the potency of S-MGB-234 and S-MGB-235 was further examined against a panel of well-characterized Gram-positive bacteria (Table 2).

**Table 2:** MICs against MDR Gram-Positive Isolates

organism	strain	S-MGB-234 (μM, (μg/mL))	S-MGB-235 (μM, (μg/mL)	ciprofloxacin(μg/mL)	meropenem (μg/mL)	vancomycin(μg/mL)
*S*. *aureus*	ATCC 9144	1.56 (1.24)	3.13 (2.13)	1	≤0.06	0.5
*S*. *aureus*	NCTC 13616	6.25 (4.98)	12.5 (8.5)	128	1–2	2
*S*. *aureus*	USA 300	3.13 (2.49)	6.25 (4.25)	64	0.25	2
*S*. *aureus*	1199B	6.25 (4.98)	6.25 (4.25)	8	≤0.06	2
*E*. *faecalis*	NCTC 775	12.5 (9.95)	12.5 (8.5)	2	1	1
*E*. *faecalis*	NCTC 12201	25–100 (19.9–79.6)	25 (17)	1	4	>128
*E*. *faecium*	NCTC 12204	6.25 (4.98)	12.5 (8.5)	1	32	>128

Both S-MGBs had broadly comparable data, with MICs between 3.13 μM and 25 μM for S-MGB-235 and 1.56 μM and 100 μM for S-MGB-234. The MICs of the control antibiotics align with the range of resistance mechanisms represented in this strain panel, none of which appeared to influence susceptibility to the S-MGBs, including fluoroquinolone resistance, which involves mutations to the topoisomerase enzymes and increased expression of efflux pumps. However, in general, both S-MGBs appeared to have higher MICs against the *Enterococci* compared to *S. aureus*.

These promising results prompted the testing of both S-MGBs against an extended panel of *S. aureus* and *Enterococcus*spp. Strains (Table 3).

**Table 3:** MICs against the Expanded Panel of MDR Gram-Positive Isolates

organism	strain	S-MGB-234 (μM, (μg/mL))	S-MGB-235 (μM, (μg/mL)	ciprofloxacin (μg/mL)	cefoxitin (μg/mL)	vancomycin(μg/mL)
MSSA	A1988	3.13 (2.49)	3.13 (2.13)	0.5	2	-
	BGW541	3.13 (2.49)	3.13 (2.13)	4	2	-
	6571	1.56 (1.24)	3.13 (2.13)	0.25	2	-
	8325	3.13 (2.49)	3.13 (2.13)	0.25	2	-
	1199	3.13 (2.49)	3.13 (2.13)	1	4	-
	19W05879	1.56 (1.24)	3.13 (2.13)	0.25	2	-
	19Y06841	3.13 (2.49)	3.13 (2.13)	≤0.125	2	-
	19W05971	3.13 (2.49)	3.13 (2.13)	0.25	4	-
	W5897	1.56 (1.24)	3.13 (2.13)	ND	4	-
MRSA	NCTC 12493	3.13 (2.49)	6.25 (4.25)	>128	16	-
	NCTC 13142	3.13 (2.49)	3.13 (2.13)	4	64	-
	NCTC 13277	6.25 (4.98)	12.5 (8.5)	128	>128	-
	19 × 19643	3.13 (2.49)	3.13 (2.13)	2	32	-
	19w05899	1.56 (1.24)	3.13 (2.13)	2	16	-
	19W05991	3.13 (2.49)	3.13 (2.13)	0.25	8	-
	19 × 19739	3.13 (2.49)	3.13 (2.13)	≤0.125	64	-
	X24051	1.56 (1.24)	3.13 (2.13)	ND	64	-
*E*. *faecalis*	ATCC 33186	>100 (>79.6)	25 (17)	ND	-	8
	ATCC 51299	>100 (>79.6)	100 (68)	ND	-	128
	ATCC 51188	>100 (>79.6)	100 (68)	ND	-	8
	NCTC 12697	12.5 (9.95)	12.5 (8.5)	4	-	16
*E*. *faecium*	ATCC 19434	6.25 (4.98)	6.25 (4.25)	8	-	4
	ATCC 35667	6.25 (4.98)	12.5 (8.5)		-	4
	ATCC 700221	6.25 (4.98)	12.5 (8.5)	>128	-	>128

Against *S. aureus*, both compounds showed similar MICs, typically between 1.56 μM and 6.25 μM, across the strains tested, and there was no difference between MSSA and MRSA strains; MRSA being defined by EUCAST as having an MIC of >4 μg/mL to cefoxitin. Considering the MICs against strain 1199 (Table 3) and strain 1199B, with NorA constitutively overexpressed in the latter strain (Table 2), there is no evidence of efflux liability mediated through NorA for either compound.^28^

Activity against *Enterococcus faecium* strains ranged from 6.25 to 12.5 μM, irrespective of ciprofloxacin or vancomycin susceptibility. However, there were strain to strain differences in both compounds’ activity for 3 of 4 *E. faecalis* strains tested, with MICs close to or beyond the range tested (>or = 100 μM). The exception being NCTC 12697 where MICs were 12.5 μM for both S-MGB-234 and S-MGB-235. Although VRE strains are included for both species in the current analysis, with no clear impact on susceptibility to S-MGB-234 and S-MGB-235, the observed differential susceptibility is the opposite to commonly used antibiotics, such as vancomycin and ampicillin, which have generally been shown to be lower in *E. faecalis* than in *E. faecium*.^29^ Possible explanations could include indirect mechanisms, such as differences in permeability between the two species, different complements of efflux pumps or direct factors affecting the target binding of MGBs to the bacterial DNA (see Fig. 1).

**Figure 1:**
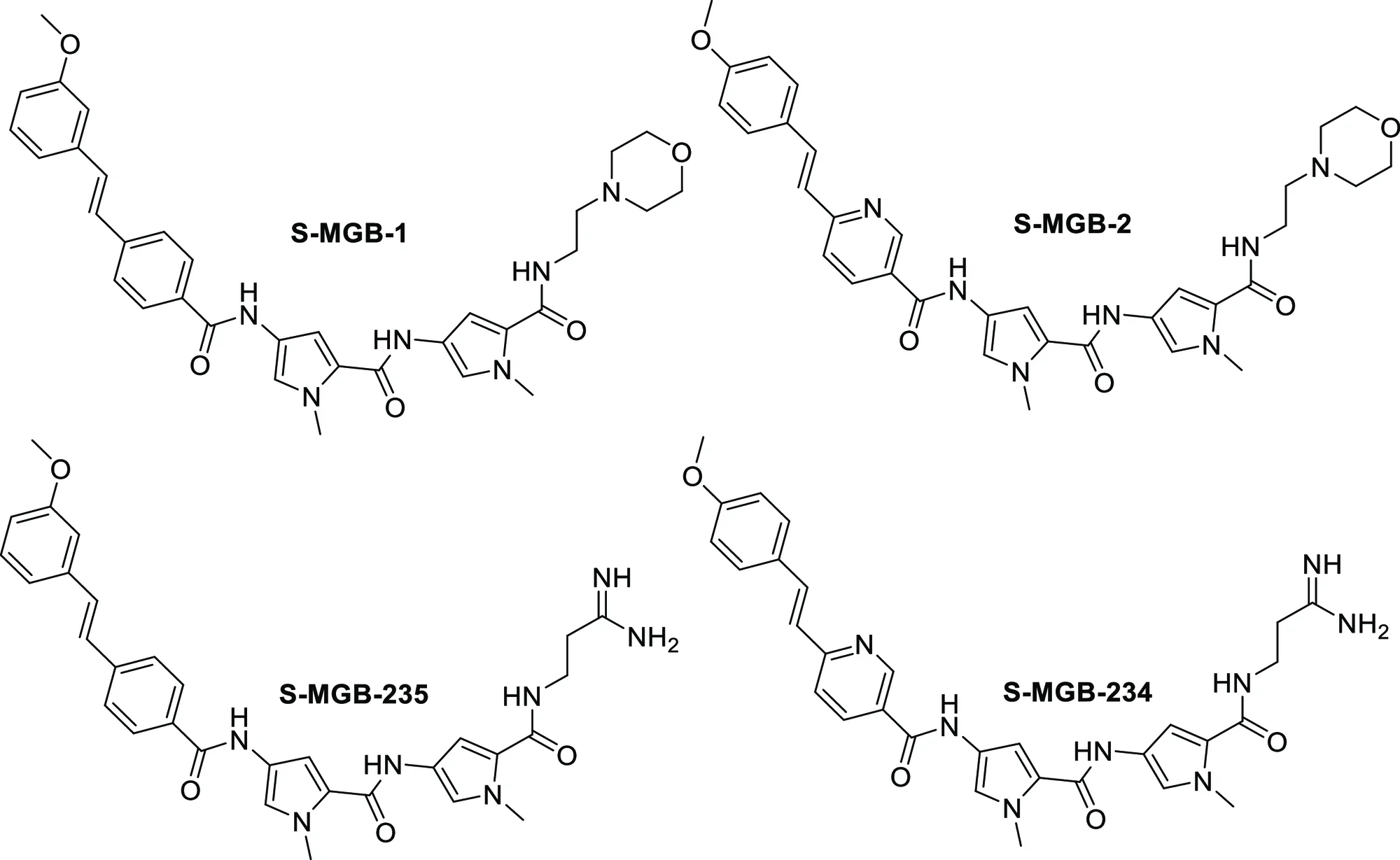
Structures of S-MGB under study in this work, specifically S-MGB-1, S-MGB-2, S-MGB-234, and S-MGB-235.

Given the generally better activity against *S. aureus* for both S-MGB-234 and S-MGB-235, time–kill assays were run at 4xMIC, for USA300 and NCTC 13616 and viable cell counts measured at 0, 1, 2, 4, 6, and 24 h (Figures 1 and 2). At 6 h, both compounds were bacteriostatic with a reduction in viable count at or below the 3-log definition used to assess bactericidal vs bacteriostatic effects. After 24 h, the viable count for NCTC 13616 was reduced to below the limit of the detection of the assay, for both compounds, suggesting that over longer time periods, the compounds become bactericidal in this strain. This was not the case for USA 300, where although there was an approximate 2-fold log reduction by 24 h, there remained a significant viable count (around 7 to 10,000 colonies per mL), not bringing it below the 3-log reduction threshold for classification as bactericidal. The control antibiotic gentamicin was bactericidal against NCTC 13616 by 4 h and against USA 300 by 6 h.

**Figure 2:**
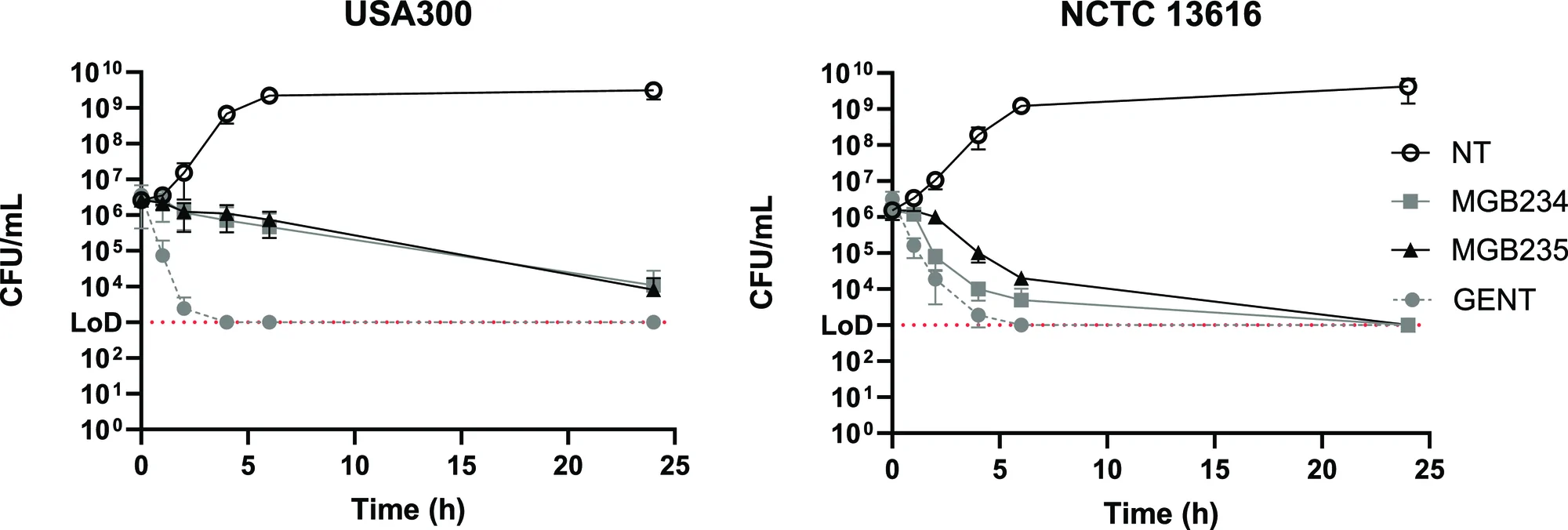
Time–kill curves of S-MGBs and the control antibiotic Gentamicin demonstrate the mode of action of the compounds. S-MGBs demonstrate bacteriostatic action against USA300 and a slow bactericidal action against NCTC 13616.

### Amidine Tail Group S-MGBs, S-MGB-234 and S-MGB-235, Bind to dsDNA

Given that S-MGBs are defined as DNA MGBs and that replacement of the morpholine tail group with an amidine can alter DNA-binding behavior in other anti-infective programs, we next investigated whether S-MGB-234 and S-MGB-235 retained the characteristic DNA-binding properties of the S-MGB class. S-MGBs from various other drug discovery programs have been established as strongly binding to double-stranded DNA (dsDNA), using two orthogonal methods: native mass spectrometry using short AT-rich dsDNA oligomers and a thermal shift analysis of genomic DNA (gDNA).^13^ Herein, we similarly assessed the amidine tail containing S-MGB-234 and S-MGB-235, along with the morpholine containing S-MGB-2 and S-MGB-1.

Native mass spectrometry experiments were carried out using a short, self-complementary DNA oligo with an AT-rich binding site, 5′-CGCATATATGCG-3′, which has been used extensively across the S-MGB program (Figure 3, Panel A). All four S-MGBs were shown to bind as a dimer [DS+2M], in charge states 5– and 4–, with no evidence of binding as a monomer [DS+1M]. This is in line with the parent molecule, distamycin, and S-MGBs from other drug discovery programs, which also bind to AT-rich dsDNA oligos as dimers.^13,19,20,25^

**Figure 3:**
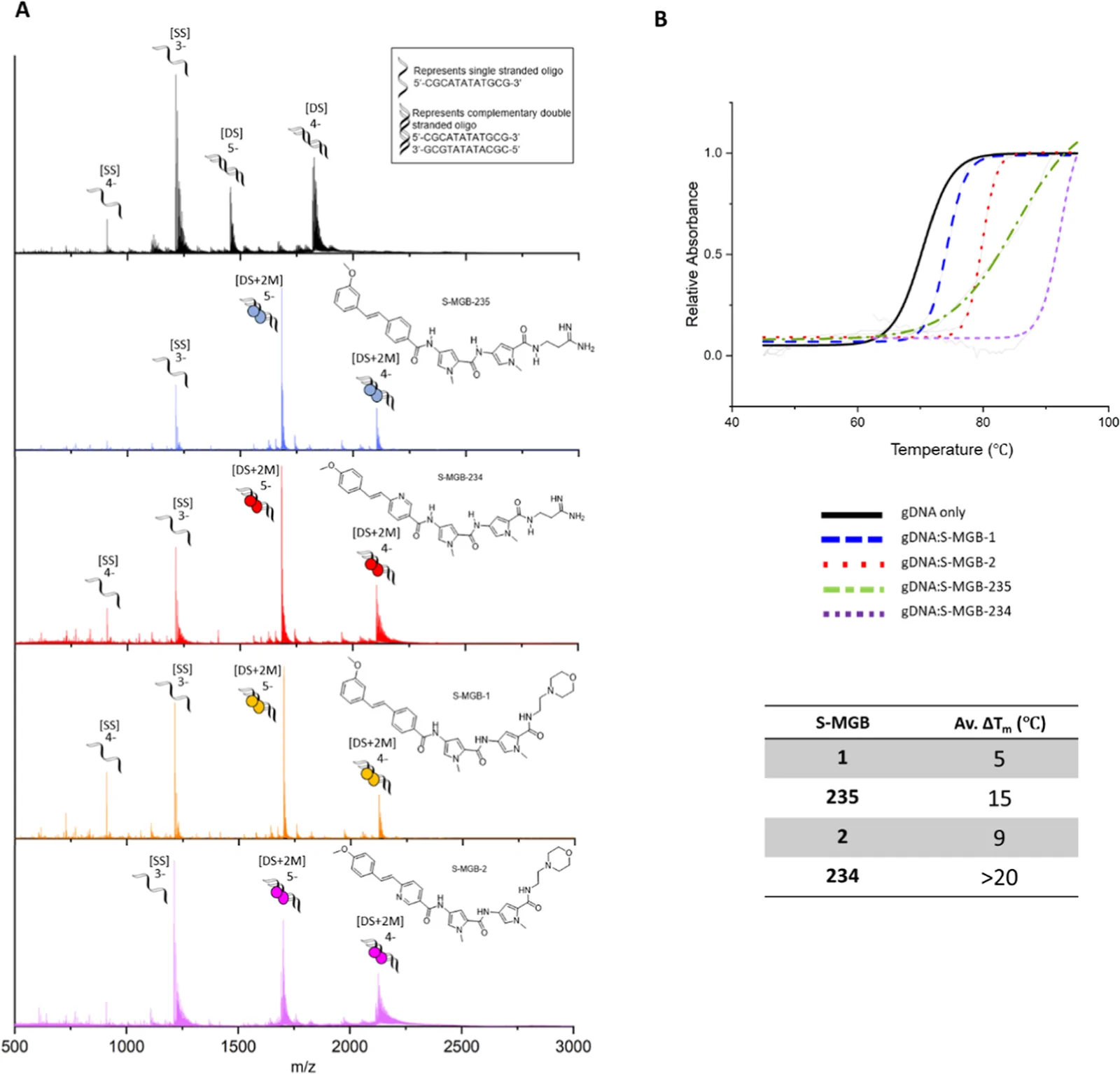
Panel A. nESI-MS characterization of S-MGBs binding to double-stranded DNA oligo 5′-CGCATATATGCG-3′. 9 μM DNA (100 μM KCl, 1% DMSO) sprayed from ammonium acetate (150 mM, pH 7) in the absence (**top**) and presence (**lower 4**) of 100 μM S-MGBs. Each subpanels shows the following: top, single-stranded DNA (denoted [SS]) was present in charge states 4– and 3–, and double-stranded DNA (denoted [DS]) was present in charge states 5– and 4–. Lower 4, [SS] was present in charge states 3– and 4–. Each [DS] molecule bound 2xS-MGB molecules (denoted [DS+2M]) and was present in charge states 5– and 4–. Expected and measured masses of each species are provided in Tables S1–S4. Panel B. Thermal melt analysis of S-MGBs bound to gDNA including the exemplar melt curve from one experimental repeat, fitted with a Boltzmann distribution (A) and computed melting temperatures for *N* = 3 experiments, with all values within ±1 °C (B).

To probe the relative strengths of the interaction between the S-MGBs and dsDNA, we also carried out a DNA thermal shift assay using a model gDNA extracted from salmon, again extensively used in the S-MGB program (Figure 3, Panel B). Comparing S-MGBs that differ only in their headgroup, S-MGB-1 vs S-MGB-2 and S-MGB-235 and S-MGB-234, indicates that the headgroup in S-MGB-2 and S-MGB-234 likely contributes to these compounds’ higher Δ*T*_m_ and stronger interaction with dsDNA. This could be due to the pyridyl group in this headgroup being able to interact with DNA through an additional hydrogen bond compared to the phenyl in the headgroup of S-MGB-1 and S-MGB-235. The Δ*T*_m_s of S-MGB-1 vs S-MGB-235 and S-MGB-2 vs S-MGB-234 (Figure 3, Panel B), with the latter in each pair having a higher Δ*T*_m_, strongly suggest that the change from the morpholine tail group to the amidine tail group increases the strength of interaction with dsDNA. This could be explained by the permanently positively charged amidine, at physiological pH, being better able to interact with the phosphate backbone of DNA. The DNA-binding studies were included to establish that S-MGB-234 and S-MGB-235 retain the characteristic DNA-binding behavior observed across the wider S-MGB platform. While these experiments demonstrate strong interactions with DNA in vitro, they do not directly establish DNA as the primary intracellular target responsible for antibacterial activity. Nevertheless, the results are consistent with a DNA-directed mechanism previously reported for related S-MGBs. The lack of a simple relationship between DNA-binding strength and antibacterial potency suggests that additional factors, including bacterial uptake and intracellular accumulation, are likely to contribute significantly to activity.

### Reduced Susceptibility Emerges More Slowly for Amidine Tail Group S-MGBs than for Gentamicin, and Resistance Isolates Are Not Cross-Resistant to Morpholine Tail S-MGBs

Noting that the structural modification to the amidine tail group appeared to be favorable in the antibacterial context in terms of in vitro potency and cytotoxicity, we were next interested in considering these S-MGB’s propensity to resistance generation while also determining any mechanisms of resistance. Triplicate wells containing NCTC 13616 were serially passaged in the presence of S-MGB-234, S-MGB-235, or the control antibiotic gentamicin (Figure 4). MICs were measured each day, and the culture at 0.5xMIC was passaged into new MIC plates. After 20 days, the MIC of the remaining culture was measured.

**Figure 4:**
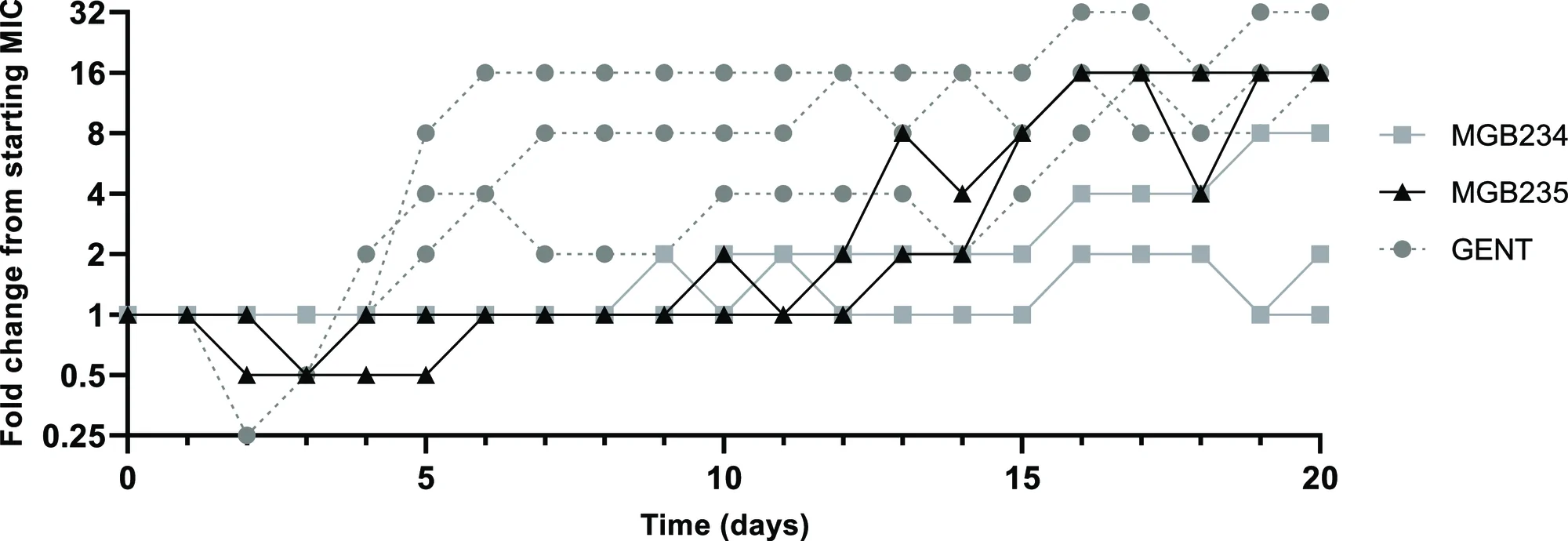
Emergence of resistance through serial passaging of S-MGBs and gentamicin in NCTC 13616.

In 2 of 2 replicates (the third replicate was contaminated), NCTC 13616 showed elevated MICs after adaptation to S-MGB-235, while just 1 of 3 replicates showed increased MICs for S-MGB-234 (the remaining 2 wells showing no significant change after 20 days serial passage) (Table 3). All gentamicin replicates (3/3) showed increased MICs to gentamicin, with resistance emerging much earlier in the passaging at days 4–7, compared to at days 13–18 for the S-MGBs. To ensure any subsequent analysis was carried out with stably resistant isolates, the populations were passaged 10 times on solid media in the absence of selection. The MICs of single colonies were rechecked, with the S-MGB-234-adapted strain showing an 8-fold increase in MIC (6.25 to 50 μM), while the two S-MGB-235-adapted strains also showed an 8-fold increase (12.5 to 100 μM). The gentamicin-adapted strains showed increases of 8–16-fold (2 to 16–32 μg/mL). Under the serial passaging conditions employed, reduced susceptibility emerged later for both S-MGBs than for gentamicin. However, this is more apparent for S-MGB-234 than for S-MGB-235, as its resistance emerges less quickly, and also in only 1 of 3 replicates.

Whole-genome sequencing analysis was performed on the stable mutants isolated from the passaging experiment (Table 4). For the single S-MGB-234 adapted strain, mutations were observed in the MFS family efflux pump, NorA, and the cell division regulator CozEb. Although we cannot be sure of the effect of the first mutation (NorA), the 5bp deletion in CozEb is likely to result in an inactive protein. CozEb mutations were also identified in isolates from one of the two wells adapted to S-MGB-235, together with mutations in the methicillin resistance protein FmtA. In both cases, mutations were identified that would generate frameshift mutations in the two protein coding sequences. SNPs were identified in FmtA in isolates from the second MGB235 well.

**Table 4:** Increase in MIC Following Adaptation of NCTC 13616 to S-MGB Compounds and the Mutations Arising from This Generation of Resistance^a^

	MICs (μM)				Mutations in selected genes of interest			
Adaptation to	WT	day 20	P10	fold change	MFS transporter (NorA) ER16_03325	methicillin resistance protein FmtA ER16_04685	cell elongation protein CozEb ER16_06300	other mutations
S-MGB-234	6.25	12.5	n/a	-	-	-	-	-
		6.25	n/a	-	-	-	-	-
		>25	50	8	A162 V	-	5bp insertion after n165	ER16_05710 phosphatidate cytidylyltransferase ***M110I***; ER16_04085 NAD(P)/FAD-dependent oxidoreductase ***1bp insertion after n872***; ER16_02560 DNA-directed RNA polymerase subunit beta ***R495C***, ***D1046E***
S-MGB-235	12.5	>100	100	8	-	Insertion 2nt (AT) after n869	Deletion 1nt n786	ER16_05710 phosphatidate cytidylyltransferase ***G118D***; ER16_04820 phosphoenolpyruvate-protein phosphotransferase ***Q368STOP***; ER16_10805 DNA-directed RNA polymerase subunit delta ***insertion 1nt*** (***A***) ***after n332***
		>100	100	8	-	V335F	-	ER16_04085 NAD(P)/FAD-dependent oxidoreductase— ***Deletion 18nt after n141***
GENT	2	32	16	8	-	-	-	ER16_05880 (dimethylallyl)adenosine tRNA methylthiotransferase ***S241R***; ER16_07635 shikimate kinase ***Deletion 1nt n296***
		>32	32	16	-	-	-	ER16_04970 heme A synthase (CtaA) ***G313D***; ER16_04085 NAD(P)/FAD-dependent oxidoreductase ***G363D***; ER16_04370 SpxA ***T11I***; ER16_09645 cysteine protease staphopain A ***P21S***; ER16_07410 transcriptional regulator (SrrA) ***1bp deletion n178***
		32	16	8	-	-	-	ER16_04970 heme A synthase (CtaA) ***G313D***; ER16_04630 1,4-dihydroxy-2-naphthoyl-CoA synthase ***G120S***; ER16_02585 FusA ***P478S***; ER16_09645 cysteine protease staphopain A ***P21S*** ER16_06940 ubiquinone biosynthesis methyltransferase UbiE ***1bp deletion n63***

a Genes associated with known resistance profiles to antibiotics (NorA, FmtA, and CozEb) are shown in the table. WT = wild-type. Day 20 = MIC at the end of the passaging experiment. P10 = MIC of isolates passaged an additional 10x without a selective agent. Fold change = change in MIC between wild-type and stably adapted isolates (P10). n/a not applicable as no elevated resistance.

Other mutations were observed in each of the isolates from the adaptation experiments, with SNPs in phosphatidate cytidylyltransferase (ER16_05710) and indels in NAD­(P)/FAD-dependent oxidoreductase (ER16_04085) also shared between at least two S-MGB-adapted isolates. In contrast, NCTC 13616 adapted to gentamicin, as a control, showed no mutations in any of the genes identified above, suggesting that the mutations have arisen due to adaptation to S-MGB234/S-MGB-235, even if not all of the shared mutations are responsible for the identified increase in resistance. Only FusA in the Gent-c population has previously been associated with gentamicin resistance in *S. aureus* but appeared as a compensatory mutation in the presence of other primary mechanisms of resistance (such as hemin or menaquinone synthetic pathways).^30^

To understand if any of these mutations were likely to mediate cross-resistance to other antibiotics or S-MGBs studied in this project, additional MIC determinations were carried out (Table 5). We included both the control compounds S-MGB-1 and S-MGB-2, which are the morpholine tail analogues of S-MGB-235 and S-MGB-234, respectively. Additionally, we included MGB-BP-3 which is an S-MGB that has successfully completed phase II clinical trials for *C. difficile*-associated disease, and it also contains a morpholine tail.^13^

**Table 5:** Cross-Resistance of S-MGB-Adapted NCTC 13616 Strains to Other S-MGBs (μM) and Antibiotics (μg/mL) (CIP; Ciprofloxacin, VAN; Vancomycin, GENT; Gentamicin, LIN; Linezolid, OXA; Oxacillin, and CXT; Cefoxitin)^a^

adaptation to	S-MGB-234	S-MGB-235	MGB-BP-3	S-MGB-1	S-MGB-2	CIP	VAN	GENT	LIN	OXA	CXT
none	12.5	12.5	0.39	6.25	3.12	64	4	4	2	32	16
S-MGB-234	100	>100	0.195	3.12	0.78	32	8	4	2	128	128
S-MGB-235	25	>100	0.78	6.25	1.25–3.12	32→128	4	4	2	8	8
	25	>100	0.195	6.25	1.56	>128	4	8	2	64	64

a The mutations in the S-MGB-234-adapted strain give cross-resistance to S-MGB-235 (≥16-fold increase in MIC), but the reverse is less apparent with only a 2-fold increase in MIC for S-MGB-234 in strains adapted to S-MGB-235.

None of the adapted strains showed any increase in MIC for the morpholine tail group containing S-MGBs, S-MGB-1, S-MGB-2, or MGB-BP-3, suggesting that the mechanisms of resistance observed here are not able to confer cross-resistance to these compounds. This, coupled with the observed cross-resistance between S-MGB-235 and S-MGB-234, would suggest that the resistance mechanisms are governed specifically by the tail group moiety in these S-MGBs.

Interestingly, the S-MGB-234-adapted strain showed an increase in the MIC for both cefoxitin and oxacillin (4- and 8-fold, respectively), suggesting that deletion of CozEb and/or mutations in the efflux pump NorA may affect activity of these β-lactam antibiotics.

In the S-MGB-235-adapted strains, we see a difference in susceptibility linked to different mutations and with differences observed for the two beta-lactams. Strains with frameshift mutations in both FmtA and CozEb showed a 4-fold reduction in the MIC for oxacillin but no significant reduction in MIC for cefoxitin. Conversely, strains with V335F mutations in FmtA gave a 4–8-fold increase in MIC for cefoxitin and a ≥ 8-fold increase in MIC to oxacillin in one of the strains. A ≥ 4-fold increase in MIC to ciprofloxacin, a fluoroquinolone antibiotic, was also observed in the two strains carrying the FmtA V335F mutation. Although this mutation has not been previously linked to FQ resistance, this may suggest that differences in growth characteristics and/or cell wall permeability also affect antibiotic susceptibility in these strains.

FmtA is a penicillin-recognizing protein with novel hydrolytic activity toward the ester bond between DAla and the backbone of teichoic acids.^31^ The V335F mutation identified in some of the S-MGB-235 adapted strains lies adjacent to one of the residues forming the putative substrate binding domains of FmtA. This may modify the function of the enzyme, and strains with this mutation also have elevated MICs for the two β-lactam substrates oxacillin and cefoxitin. FmtA has been recognized previously as an enzyme capable of modulating the function of beta-lactam antibiotics^32,33^ mediated through interactions with cell wall teichoic acids,^34^ and FmtA itself may represent a novel antibiotic target.^35^ CozEb may also play a role in modification or regulation of peptidoglycan biosynthesis, specifically in relation to the cell division cycle in *S. aureus*.^36^ Deleterious mutations in CozEb are not lethal, given the presence of a second related gene, CozEa, but the two proteins appear also to have specialized functions with the former being involved in regulating peptidoglycan synthesis around the cell septum during cell division. Mutations in CozEb result in smaller cell size, and this is perhaps reflected in this study with notably smaller colonies observed in all of the MGB-adapted strains.

Since mutations arose in FmtA and CozEb in response to passage in the presence of both S-MGB-234 and S-MGB-235, we next assessed whether these could be additional direct targets for their action, in addition to the proposed DNA-binding-mediated effects. This was achieved by measuring activity in transposon mutant strains compared to the wild-type parent strain, USA300 (Table 6).^37^

**Table 6:** MICs of Antibiotics (μg/mL) and S-MGBs (μM) against USA300 Transposon Mutant Strains from the Nebraska Centre for Staphylococcal Research

	transposon mutant	OXA	CXT	S-MGB-234	S-MGB-235
**USA300 WT**	-	16	32	3.125	6.35
**fmtA**	NR-47565 SAUSA300-0959	**1**	16	6.25	6.25
**cozEB**	NR-47322 SAUSA300-1254	**1**	32	6.25	6.25
**fmtC**	NR-47902 SAUSA300-1255	**0**.**5**	**4**	3.125	6.25

Although there were noticeable reductions in the MIC for oxacillin (in all three Tn strains), this was not the case for cefoxitin, where a reduction in MIC was only observed in the case of fmtC, which might reflect differences in PBP binding affinities. No significant increase in MIC was observed in any of the mutant strains for the S-MGBs, suggesting that these proteins are not direct targets of these compounds. Instead, we speculate that mutations in these proteins observed in the S-MGB-adapted strains might confer a protective effect through their teichoic acid- or peptidoglycan-related functions by reducing the intracellular accumulation of the S-MGBs.^36,38^

A clear mechanism is challenging to construct for CozEB. However, the reduction of FmtA’s esterase activity could increase the positive charge density on the cell surface due to an increase in the number of positively charged d-alanine esters functionalizing the otherwise negatively charged teichoic acids.^34^ Indeed, alteration of cell surface charge density is known to affect antibacterial susceptibility for cationic antimicrobial peptides.^39,40^ This could explain the difference in the fold change of MICs for the S-MGBs considered in this work. The amidine tail will be almost entirely positively charged a physiological pH, conferring a greater positive charge density to S-MGB-234 and S-MGB-235 compared to S-MGB-1, S-MGB-2 and MGB-BP-3, bearing weakly basic morpholine tails. The more positive cell surface charge density of the FmtA mutant strains could repel the amidine tail containing S-MGBs, reducing the activity of these compounds. The activity of morpholine tail-containing S-MGBs would be less affected by an increase in cell surface positive charge density, in line with the limited change in the activity of these compounds against the FmtA mutant strains. Indeed, Stokes et al. (2019) previously demonstrated that another class of amidine-containing MGB can interact with the cell envelope, specifically the LPS layer of Gram-negative bacteria, as part of their mechanism of action.^11^ Here, we tentatively suggest a similar interaction with the cell envelope of Gram-positive bacteria.

Building upon the resistant strains generated against S-MGB-234 and S-MGB-235 using the 20-passage technique, we next attempted to generate resistance in a wider range of *S. aureus* strains. Additionally, we also included S-MGB-1 and S-MGB-2 in an attempt to compare resistance emergence between the morpholine and amidine tail group compounds (Tables 7 and S6). This was conducted following a higher-throughput method, where each strain was incubated with increasing concentrations, from 0.25xMIC up to 4xMIC, of each S-MGB over 5 passages for 24 h each.

**Table 7:** A Selection of *S. aureus* Isolates Were Passaged with Increasing Concentrations of S-MGB-1, S-MGB-2, S-MGB-234, and S-MGB-235^a^

					mutations		
adaptation	S-MGB-1	S-MGB-2	S-MGB-234	S-MGB-235	gene	mutation	function
NCTC 13616							
**13616**-**S**-**MGB**-**1**	2	0	0	0	-	-	-
**13616**-**S**-**MGB**-**2**	0	2	0	0	-	-	-
**13616**-**S**-**MGB**-**234**	0	0	2	2	-	-	-
**13616**-**S**-**MGB**-**235**	0	2	2	0–8	-	-	-
**ATCC 9144**							
**9144**-**S**-**MGB**-**1**	0	0	0	0	-	-	-
**9144**-**S**-**MGB**-**2**	4	8	4	2	NorA	Promoter Region	MFS transporter (AH5611_000651)
**9144**-**S**-**MGB**-**234**	2	4	2	2	-	-	-
**9144**-**S**-**MGB**-**235**	2	2	4	2	-	-	-
**USA300**							
**USA300**-**S**-**MGB**-**1**	2	2	0	2	-	-	-
**USA300**-**S**-**MGB**-**2**	8	8	2	4	CozEB ktrA	I191N	cell elongation protein (AH5611_001230)
					NorA	E76G	Ktr system potassium uptake protein A (AH5611_000940)
						A362T	MFS transporter (AH5611_000651)
**USA300**-**S**-**MGB**-**234**	-	-	-	-	-	-	-
**USA300**-**S**-**MGB**-**235**	2	0	2	4	CozEB ktrA	I191N	cell elongation protein (AH5611_001230)
					NorA	E76G	Ktr system potassium uptake protein A (AH5611_000940)
						A362T	MFS transporter (AH5611_000651)
**1199B**							
**1199B**-**S**-**MGB**-**1**	2	2	0	0	-	-	-
**1199B**-**S**-**MGB**-**2**	4	4	0	0	gmuF	C36F	mannose-6-phosphate isomerase (AH5611_002003)
					walk	D496G	sensor protein kinase (AH5611_000021)
					AgrA	Q61P	accessory gene regulator A (AH5611_001892)
**1199B**-**S**-**MGB**-**234**	2	2	0	2–8	ProS	G438S	proline--tRNA ligase (AH5611_001113)
					MenG_2	1 bp del n63	demethylmenaquinone methyltransferase (AH5611_001344) hypothetical protein (AH5611_000022)
					-	21 bp del after 101	
**1199B**-**S**-**MGB**-**235**	4	4	2	4	MenG_2 yxeP_4	Q164STOP	demethylmenaquinone methyltransferase (AH5611_001344) putative hydrolase (AH5611_002187)
					WalK	I31N	sensor protein kinase (AH5611_000021)
					AgrA	D235N	accessory gene regulator A (AH5611_001892)
					RpoB	Q61P	DNA-directed RNA polymerase subunit betaa (AH5611_000502)
						S143Y	

a The fold-change in MIC is recorded between the wild-type and the post-adaptation strains for each S-MGB. Where no data is recorded, strain did not survive the passaging. Red = some level of resistance/tolerance, due to a >4-fold increase in MIC. Table S6 presents the same MICs.

All strains except USA300-S-MGB-234 grew up to 4xMIC, but when isolates were passaged on solid media in the absence of selection and retested by MIC, resistance did not arise, or was not stable, in most cases (yellow shaded boxes in Table 7). Higher MICs were only observed for the compound the strain was adapted to in 6 instances out of 16, and only for S-MGB-235 and S-MGB-2. In these cases, the maximum fold change in MIC was 8-fold. Cross-resistance to the other S-MGBs was also not a common occurrence, with resistance to S-MGB-234 only observed in 9144-MGB-2- and 9144-MGB-235-adapted strains. Interestingly, reliable resistance was not detected in NCTC 13616, which is the strain previously used in the 20-passage method.

Isolates where stable resistance was observed were whole-genome-sequenced (Table 7). The detected mutations seemed to be dependent on the strain background. For all ATCC 9144- and USA300 S-MGB-resistant strains, efflux pump NorA was mutated. The 1199B strain already has upregulated NorA, so additional mutations in the efflux pump gene were unlikely to occur. The CozEb mutant detected in the 20-passaged NCTC 13616 strains was again detected here, but only in the USA300 strains. The other mutation in the USA300 strains is in ktrA, a potassium uptake protein essential for survival in low potassium conditions,^41^ and implicated in resistance to osmotic stress.^42^ The MGB-resistant 1199B strains appear to have a range of different mutations across the isolates. WalK is part of a two-component system located in the cell membrane,^43^ regulating genes involved in cell wall metabolism, virulence regulation, biofilm production, osmotic stress resistance, and antibiotic resistance.^44,45^ MenG_2 is a membrane-associated protein, which catalyzes the biosynthesis of menaquinone required for respiration, and is a major drug target in TB and NTMs.^46^

Interestingly, NorA expression did not appear to significantly impact the efficacy of the compounds in initial MIC screens (1199 vs 1199b, where 1199b has upregulated NorA, Tables 2 and 3), but the *NorA* gene was consistently implicated in the generation of resistance to S-MGBs, alongside other mutations. Whether these mutations result in altered efflux is unclear, but although there was no significant reduction in MICs (i.e., 4-fold or higher) in 1199b compared to 1199, there was a consistent 2-fold reduction. This may imply that efflux of compounds through NorA has a small effect on the activity of the compounds but that it is not a primary resistance mechanism.

## Conclusion

We evaluated two amidine tail group-containing S-MGBs, S-MGB-234 and S-MGB-235, known to be potent and tolerated in vivo as antiparasitics, for their antibacterial potential, along with two morpholine tail-containing comparator compounds, S-MGB-2 and S-MGB-1, respectively. Emerging thinking from the S-MGB platform is that compounds bearing amidine tails are less cytotoxic toward mammalian cells compared to morpholine tail comparators, providing a strong rationale for the investigation into S-MGB-234 and S-MGB-235. In line with most S-MGBs, these compounds did not have any measurable activity against Gram-negative ESKAPE pathogens. This is likely due to lack of sufficient intracellular concentrations, as has been demonstrated for the lead antibacterial S-MGB, MGB-BP-3, which contains a morpholine tail and has successfully completed phase II clinical trials to treat CDAD.

In this small panel of two matched molecular pairs, we did identify a small increase in MIC associated with the modification of the tail group from morpholine (S-MGB-1, S-MGB-2) to amidine (S-MGB-235, S-MGB-234), specifically in the S-MGB-2 and S-MGB-234 pair (a 4-fold increase) across both Gram-positive organisms, *S. aureus* and *E. faecalis*. Nevertheless, we found both S-MGB-235 and S-MGB-234 to have sufficient potency against a range of Gram-positive strains to warrant their further investigation. Cytotoxicity data also suggested comparable or better selectivity of these compounds compared to their morpholine tail analogues. Therefore, we suggest that modification of the S-MGB tail group to the amidine moiety is of interest in the antibacterial drug discovery space.

Interestingly, we did notice that the MICs against *E. faecalis* were in general slightly higher (≤4-fold) than those against *S. aureus*. This difference could reflect species-specific permeability,^47^ efflux systems, or intrinsic factors that alter S-MGB binding to DNA.

In *S. aureus* NCTC 13616, reduced susceptibility emerged more slowly during serial passaging with S-MGB-234 and S-MGB-235 than with gentamicin. However, the limited number of strains and replicates examined means that broader conclusions regarding resistance liability cannot yet be drawn.

Considering the mutants observed across both resistance generation experiments and their susceptibilities to S-MGBs, there is limited commonality that would suggest a resistance liability from specific and prominent resistance mechanisms. Instead, the resistance response appears to be heterogeneous and not focused on DNA-related mechanisms, except for only three instances of mutations in DNA-directed RNA polymerase subunits, across all experiments.

Importantly, where mutations have conferred low levels of resistance (maximum 8-fold) to some S-MGBs, all S-MGBs are not cross-resistant. In particular, we note that some S-MGB-234- and S-MGB-235-adapted strains are not cross-resistant to MGB-BP-3, the most developed S-MGB, and a compound of interest in the treatment of CDAD. Moreover, an analysis of cross-resistance patterns suggests that some resistance mechanisms are specific to the tail group of the S-MGB, in this case, either the strongly basic amidine or the weakly basic morpholine.

The most consistent mutations are observed in NorA, FmtA, and CozEb, which are all involved with the cell envelope. Connecting the function of NorA, an MFS transporter, or CozEb, implicated in peptidoglycan biosynthesis, to an S-MGB resistance mechanism is not obvious. However, we tentatively suggest a mechanism for FmtA.^34^ The balance of positive to negative charge in the cell envelope in Gram-positive bacteria can alter antibiotic susceptibility, which is known for various cationic antimicrobial peptides.^39^ In our case, the S-MGBs with a positively charged amidine tail, S-MGB-234 and S-MGB-235, could be more repelled by the cell envelope of the FtmA mutants, compared to the morpholine tail S-MGBs, due to the increased positive charge density on the cell surface, which explains their different susceptibility patterns.

S-MGBs are known to bind multiple AT-rich DNA sequences, and previous studies have linked this behavior to antimicrobial activity within the wider S-MGB platform. This multitargeted response has been said to give rise to a resilience to target-based resistance. The heterogeneous resistance response observed in this study, coupled with the assertion that the most common resistance mechanisms may involve a reduced intracellular accumulation of S-MGB, suggests that any resistance mechanisms do not involve a modulation of the interaction of the S-MGB with its DNA targets. Therefore, S-MGBs’ advantageous resilience to target-based resistance is maintained.

In summary, amidine tail S-MGBs are potent and selective against Gram-positive bacteria and do not appear to have major target-based resistance liabilities. Significantly, we have identified that the tail group of the S-MGB structure may dictate the specific resistance mechanisms, and this opens the possibility of exploring further structure–resistance relationships.

## Methods

### Compound Synthesis

S-MGB-1, S-MGB-2, S-MGB-235, and S-MGB-234 were prepared as previously described in ref (15). MGB-BP-3 was prepared as previously described in ref (26).

#### In Vitro Antibacterial Assay

The MIC was defined using a microbroth dilution method, based on EUCAST guidelines.^48^ Briefly, bacteria at a concentration of 1 × 10^5^ CFU/mL were incubated with doubling dilutions of S-MGB, prepared from a 10 mM DMSO stock, in a 96-well plate for 20 h at 37 °C in CAMHB. The MIC was defined as the lowest concentration of the compound to inhibit visible growth. MICs are presented as modal values from three independent biological experiments, where a range is reported; it is because a modal value could not be defined after 3 repeats.

#### HEK293 Assay

HEK293 cells, sourced from Sigma-Aldrich, UK, were cultured in 75 cm^2^ vented flasks with a seeding concentration of 1.5 × 10^4^ cells/cm^2^ in DMEM (pH 7.4). The cells were incubated at 37 °C with 5% CO_2_, and the growth medium consisted of DMEM supplemented with 10% FBS, 1% penicillin/streptomycin, 2 mM l-glutamine, 1 mM sodium pyruvate, and 4.5 g/L glucose, all obtained from Sigma-Aldrich, UK. Upon reaching 80% confluence, the cells were passaged using the 1X TrypLe express enzyme from Thermo Fisher Scientific, UK.

For viability assessment, HEK293 cells were seeded at 15,000 cells/well in 50 μL on black 96-well half-area clear-bottomed plates (Greiner, UK). Following an overnight attachment period, the cells were exposed to S-MGBs at concentrations ranging from 0.05 to 100 μM in 60 μL per well (S-MGBs were dissolved in DMSO at 10 mM). Control wells contained cells without compounds, representing 100% viability. Complete kill control involved the addition of 0.1% Triton X. After 24 h of incubation at 37 °C (humidified, 5% CO2), cell viability was determined using 10% v/v PrestoBlueTM HS Cell Viability reagent (Sigma-Aldrich, UK). The resulting reduction in Presto Blue was quantified with a Hidex Sense multimode plate reader (Lablogic, UK and Ireland) in fluorescent mode (excitation: 535/20 nm, emission: 595/10 nm).

The viability of treated samples was expressed as a percentage of control wells containing DMSO. Statistical analysis utilized mean ± SE values from three independent experiments. Data plotting was performed using Graph Pad Prism Software (version 9.00 for Windows, GraphPad Software, La Jolla, California, USA, www.graphpad.com).

#### UV–Vis DNA Thermal Melting Experiments

Salmon genomic DNA (gDNA; D1626, Sigma-Aldrich) at 1 mg/mL in 1 mM phosphate buffer (pH 7.4) containing 0.27 mM KCl and 13.7 mM NaCl (P4417, Sigma-Aldrich) was annealed at 90 °C for 10 min and left to cool to room temperature. S-MGBs at 10 mM in DMSO were diluted with the same phosphate buffer to yield a single sample with 10 μM S-MGB and 0.02 mg/mL gDNA in 1 mM phosphate buffer containing 0.27 mM KCl and 13.7 mM NaCl. Control samples containing only S-MGB or gDNA were prepared, respectively. Samples were melted at a rate of 0.5 °C/min from 45 to 90 °C with spectra recorded at 260 nm on a UV-1900 UV–vis spectrophotometer fitted with a Peltier temperature controller (Shimadzu) using LabSolutions (Tm Analysis) software. The melting temperatures (Tms) of the S-MGB:DNA complexes were determined by fitting a sigmoidal function using a Boltzmann distribution in OriginPro.

### Native Mass Spectrometry

DNA sample preparation: DNA oligonucleotide sequence 5′-CGCATATATGCG-3′ was purchased in the lyophilized form from Alpha DNA, Canada, and used without further purification; purity was assessed by NMR. 100 μM stock solutions of DNA were prepared with a 150 mM ammonium acetate solution (Fisher Scientific, Loughborough, Leicestershire, UK) and a 2 mM potassium chloride solution (Fisher Scientific, Loughborough, Leicestershire, UK). This solution was annealed at 90°C for 10 min and allowed to cool to room temperature. 10 mM S-MGB stock in 100% DMSO (Sigma-Aldrich, St. Louis, MO, USA) was diluted to a 1 mM S-MGB solution with 150 mM ammonium acetate. Final samples were prepared from this solution to yield final concentrations of 9 μM DNA, 100 μM KCl, and 100 μM s-MGB, 1% DMSO. DNA solutions containing no S-MGB included 1% DMSO and were used as controls.

Mass spectrometry measurements: Native mass spectrometry experiments were carried out on a Synapt G2Si instrument (Waters, Manchester, UK) with a nanoelectrospray ionization source (nESI). Mass calibration was performed by a separate infusion of NaI cluster ions. Solutions were ionized from a thin-walled borosilicate glass capillary (i.d. 0.78 mm, o.d. 1.0 mm, Sutter Instrument Co., Novato, CA, USA) pulled in-house to nESI tip with a Flaming/Brown micropipette puller (Sutter Instrument Co., Novato, CA, USA). A negative potential in range of 1.0 kV–1.2 kV was applied to the solution via a thin platinum wire (diameter 0.125 mm, Goodfellow, Huntingdon, UK). The following nondefault instrument parameters were used for the DNA: S-MGB-241 complex: capillary voltage of 1.4 kV, sample cone voltage of 100 V, source offset of 110 V, source temperature of 40 °C, trap collision energy of 3.0 (V), and trap gas of 3 mL/min. For DNA with no MGB present, the following parameters were changed: capillary voltage of 1.0 kV, sample cone voltage of 80 V, source offset of 95 V, and trap gas of 4.0 mL/min. Data were processed using Masslynx V4.2 and OriginPro 2021, and figures were produced using Chemdraw.

#### Time–Kill Assay

Bacteria at a starting concentration of 1 × 10^5^ CFU/mL were incubated in 3 mL of broth in glass universals in the presence of the compound at 4xMIC for 24 h at 37 °C with shaking. At time points of 0, 1, 2, 4, 6, and 24 h, small aliquots were removed. and a Mile and Misra method was performed to determine the CFU/mL. Bactericidal action is defined as a > 3-log reduction in CFU/mL compared to the starting concentration. Time–kill data are averaged from three independent biological experiments.

### Resistance Generation and Whole Genome Sequencing

Resistance to S-MGBs was generated in the laboratory following two methods, either in tubes or on 96-well plates. The tube method incubates bacteria at a starting concentration of 1 × 10^5^ CFU/mL in 3 mL of broth with doubly increasing concentrations of the compound, starting at 0.25x MIC. After 24–48 h of incubation at 37 °C with shaking, bacteria from the 0.25x MIC culture were diluted 1 in 1000 into media containing 0.5x MIC. This was continued up to 4x MIC if growth was still present. An MIC was performed on the surviving cultures to determine whether resistance had developed. This experiment was conducted as one biological experiment with three independent starter cultures.

The plate-based method passes strains in 96-well plates over a period of 20 days. Each day, an MIC plate is set up as above and incubated for 24 h. The MIC is then read, and culture from the well at 0.5x MIC is taken, diluted 1 in 1000, and used as the starting culture for the next MIC. This is repeated 20 times with the MIC recorded each day. If the MIC increases over time, then resistance has developed. This experiment was conducted as a single biological experiment for each strain. The resistant cultures from both methods were then passaged 10 times on solid agar in the absence of selective pressure, and the MIC values were retested. If resistance was still present, cultures were whole-genome-sequenced to identify the stable mutations present. Genomic DNA was extracted using the Promega Wizard Genomic DNA purification kit, and genomes were sequenced by UKHSA-GSDU on an Illumina (HiSeq 2500).^49^ Potential individual mutations were identified using Galaxy.^50^

## Supplementary Material



## Notes

Additional data on the cytotoxicity of S-MGBs, native mass spectrometry, and gDNA thermal shift assay; MICs of resistant isolates of *S. aureus* (PDF)
