# Supplementary material for: Strathclyde Minor Groove Binders Bearing Amidine Tail Groups are Effective against Gram-Positive Bacterial Pathogens
Source: ACS Infect Dis. 2026 Jul 14;12(8):2825–37. doi: 10.1021/acsinfecdis.6c00443 (PMC13488447; doi:10.1021/acsinfecdis.6c00443)
Supplement: Supplementary file 1 [file id6c00443_si_001.pdf]

S-MGBs bearing amidine tail groups are effective against Gram-positive bacterial pathogens

Charlotte K. Hind,[1] Kaveh Eskandari,[2] Leah M. C. McGee,[2] Rebecca Beveridge,[2] Louise C. Young,[3] Edward Hutchings,[1] Matthew E. Wand,[1] Melanie Clifford,[1] J. Mark Sutton[1,4] and Fraser J. Scott[2*]

[1]Research and Evaluation, UKHSA Porton Down, Salisbury, SP4 0JG, United Kingdom

[2]Department of Pure and Applied Chemistry, University of Strathclyde, Glasgow UK

[3]Strathclyde Institute of Pharmacy and Biomedical Sciences, University of Strathclyde, Glasgow UK

[4]Institute of Pharmaceutical Science, School of Cancer & Pharmaceutical Science, King's College London, Franklin-Wilkins Building, 150 Stamford Street, LondonSE1 9NH, United Kingdom

*Email: fraser.j.scott@strath.ac.uk

Supplementary Information

Page S2: 1. Cytotoxicity of S-MGBs

Page S3: 2. Native Mass Spectrometry

Page S5: 3. gDNA Thermal Shift Assay

Page S6: 4. Generation of Resistant Isolates of *S. aureus*

1. Cytotoxicity of S-MGBs

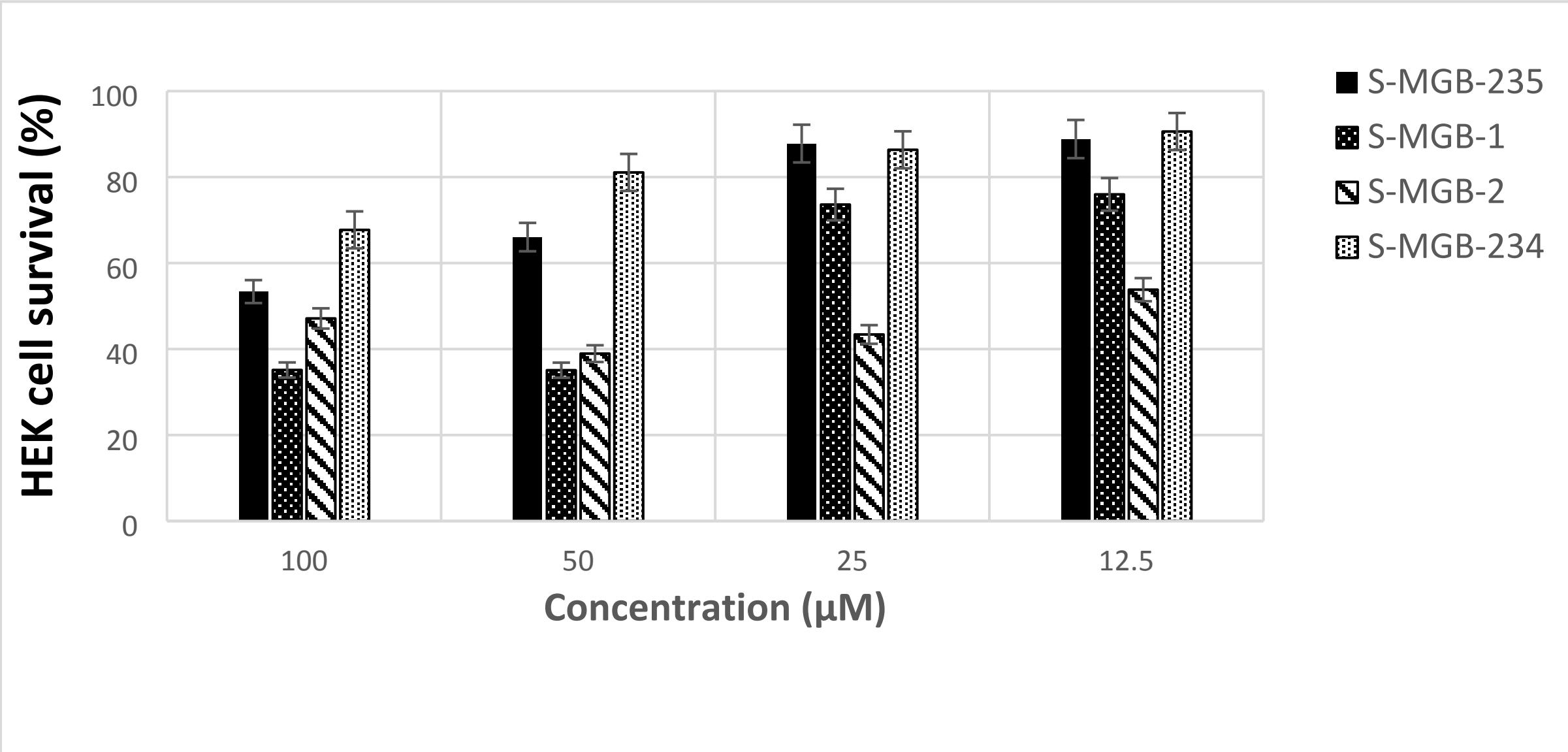

Figure S1. HEK293 cytotoxicity was screened at different concentrations (12.5, 25, 50, and 100 µM) of four S-MGBs. The data represented are the mean ±SE percentage of cell survival obtained from three independent experiments in DMEM media containing glucose. PrestoBlue™ reagent was used to assess viability of the cells after 24 hours incubation.

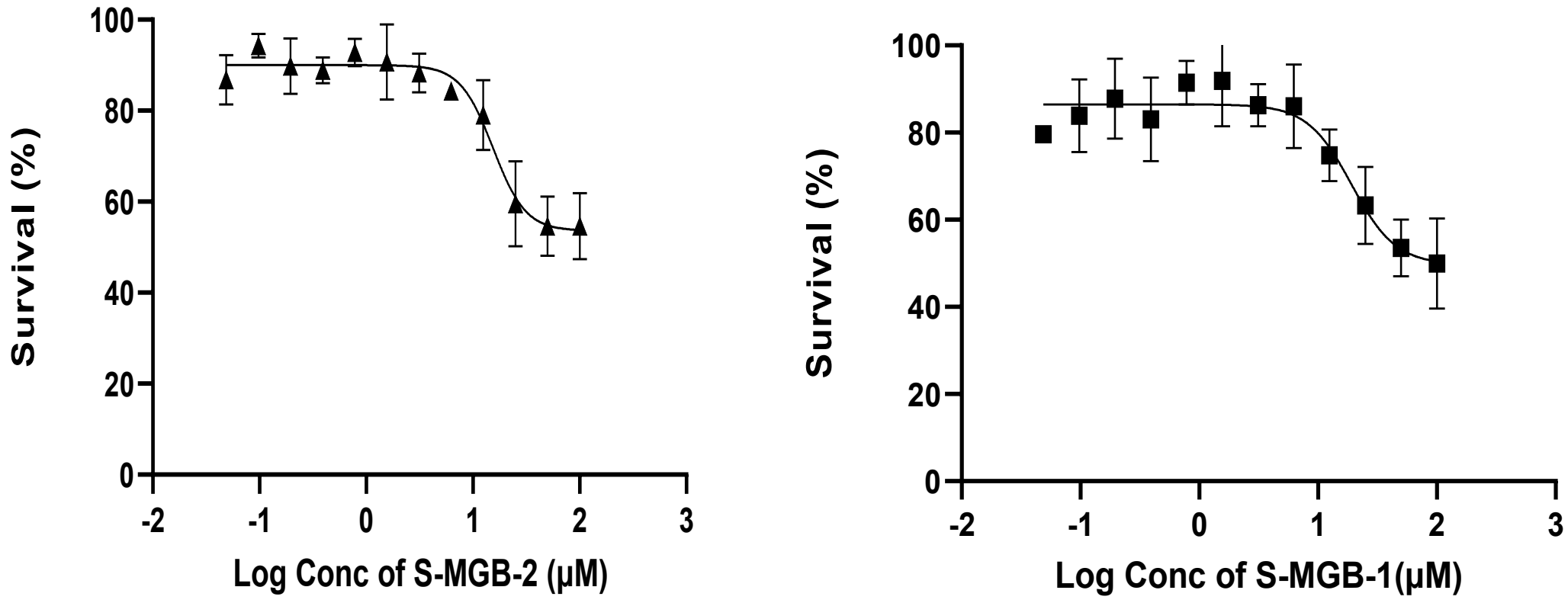

Figure S2. HEK293 cytotoxicity was determined at different concentrations (0.05-100µM) of S-MGB-1 and S-MGB-2. The data represented are the mean ± SE percentage of cell survival obtained from three independent experiments in DMEM media containing glucose. PrestoBlue™ reagent was used to assess viability of the cells after 24 hours incubation.

2. Native Mass Spectrometry

Table S1. Calculated and measured masses for each species observed in Figure S3 for DNA sequence 5'-CGCATATATGCG-3' **S-MGB-235**

Species	m/z value	Calculated mass of neutral species (Da)
**Single Stranded [SS]**	3- : 1214.1<br>4- : 910.3	(1214.1*3) + 3 = 3645.3<br>(910.3*4) + 4 = 3645.2
**Double Stranded [DS]**	4- : 1821.7<br>5- : 1457.2	(1821.7*4) + 4 = 7290.8<br>(1457.2*5) + 5 = 7291.0
Double Stranded + 2 x S-MGB-235 [DS+2M]	4- : 2105.5<br>5- : 1684.2	(2105.5*4) + 4 = 8426.0<br>(1684.2*5) +5 = 8426.0

Table S2. Calculated and measured masses for each species observed in Figure 3 for DNA sequence 5'-CGCATATATGCG-3' **S-MGB-234.**

Species	m/z value	Calculated mass of neutral species (Da)
**Single Stranded [SS]**	3- : 1214.1<br>4- : 910.3	(1214.1*3) + 3 = 3645.3<br>(910.3*4) + 4 = 3645.2
**Double Stranded [DS]**	4- : 1821.7<br>5- : 1457.2	(1821.7*4) + 4 = 7290.8<br>(1457.2*5) + 5 = 7291.0
***Double Stranded + 2 x S-MGB-234 [DS+2M]**	4- : 2106.0<br>5- : 1684.6	(2106.0*4) + 4 = 8428.0<br>(1684.6*5) + 5 = 8428.0

Table S3. Calculated and measured masses for each species observed in Figure 3 for DNA sequence 5'-CGCATATATGCG-3' **S-MGB-1.**

Species	m/z value	Calculated mass of neutral species (Da)
**Single Stranded [SS]**	3- : 1214.1<br>4- : 910.3	(1214.1*3) + 3 = 3645.3<br>(910.3*4) + 4 = 3645.2
**Double Stranded [DS]**	4- : 1821.7<br>5- : 1457.2	(1821.7*4) + 4 = 7290.8<br>(1457.2*5) + 5 = 7291.0
Double Stranded + 2 x S-MGB-1 [DS+2M]	4- : 2127.0<br>5- : 1701.4	(2127.0*4) + 4 = 8512.0<br>(1684.6*5) +5 = 8512.0

Table S4. Calculated and measured masses for each species observed in Figure 3 for DNA sequence 5'-CGCATATATGCG-3' **S-MGB-2.**

Species	m/z value	Calculated mass of neutral species (Da)
**Single Stranded [SS]**	3- : 1214.1<br>4- : 910.3	(1214.1*3) + 3 = 3645.3<br>(910.3*4) + 4 = 3645.2
**Double Stranded [DS]**	4- : 1821.7<br>5- : 1457.2	(1821.7*4) + 4 = 7290.8<br>(1457.2*5) + 5 = 7291.0
Double Stranded + 2 x S-MGB-2 [DS+2M]	4- : 2127.5<br>5- : 1701.8	(2127.5*4) + 4 = 8514.0<br>(1701.8*5) +5 = 8514.0

3. gDNA Thermal Shift Assay

Table S5. Thermal shift assay of gDNA and gDNA:S-MGB complexes calculated from fitted Boltzmann distributions using OriginPro 2021. Values in bold are associated with the representative data present in Figure 3.

S-MGB	RUN	gDNA T_m (℃)	gDNA:S-MGB Complex T_m (℃)	Average T_m Difference (°C)
1	1	69.82	74.23	
1	2	69.82	74.21	
1	3	70.1	75.01	
1	4	70.51	75.35	4.64
235	1	69.30	84.62	
235	2	70.85	84.30	
235	3	70.00	85.34	
235	4	69.84	**85.08**	14.84
2	1	**69.73**	79.63	
2	2	70.37	**79.83**	
2	3	70.28	77.52	
2	4	69.97	77.44	8.52
234	1	69.37	92.21	
234	2	69.14	**92.54**	
234	3	69.58	> 90	
234	4	68.98	> 90	> 20.73

4. Generation of Resistant Isolates of *S. aureus*

Table S6. A selection of *S. aureus* isolates were passaged with increasing concentrations of S-MGB-1, S-MGB-2, S-MGB-234 and S-MGB-235. The MICs are presented for the wild-type and the post-adaptation strains for each S-MGB. Where no data is recorded, strain did not survive the passaging. Red = some level of resistance/tolerance, due to ≥4-fold increase in MIC. Table 6 presents the same data as fold-change.

	S-MGB-1	S-MGB-2	S-MGB-234	S-MGB-235
**NCTC 13616**	3.12	3.12	6.25	12.5
**13616-S-MGB-1**	6.25	3.12	6.25	6.25-12.5
**13616-S-MGB-2**	6.25	6.25	6.25	6.25
**13616-S-MGB-234**	6.25	3.12	12.5	25
**13616-S-MGB-235**	6.25	6.25	12.5	12.5-100
**ATCC 9144**	1.56	0.78	1.56	3.12
**9144-S-MGB-1**	1.56	0.78	1.56	3.12
**9144-S-MGB-2**	6.25	6.25	6.25	6.25
**9144-S-MGB-234**	3.12	3.12	3.12	6.25
**9144-S-MGB-235**	3.12	1.56	6.25	6.25
**USA300**	1.56	0.78	3.12	3.12
**USA300-S-MGB-1**	3.12	1.56	3.12	6.25
**USA300-S-MGB-2**	12.5	6.25	6.25	12.5
**USA300-S-MGB-234**	-	-	-	-
**USA300-S-MGB-235**	3.12	0.78	6.25	12.5
**1199B**	3.12	1.56	6.25	6.25
**1199B-S-MGB-1**	6.25	3.12	6.25	6.25
**1199B-S-MGB-2**	12.5	6.25	6.25	6.25
**1199B-S-MGB-234**	6.25	3.12	6.25	12.5-50
**1199B-S-MGB-235**	12.5	6.25	12.5	25
